# Biological effects of tremolite.

**DOI:** 10.1038/bjc.1982.61

**Published:** 1982-03

**Authors:** J. C. Wagner, M. Chamberlain, R. C. Brown, G. Berry, F. D. Pooley, R. Davies, D. M. Griffiths

## Abstract

**Images:**


*Br. J. Cancer* (1982) **45**, 352

### BIOLOGICAL EFFECTS OF TREMOLITE

J. C. WAGNER, M. CHAMBERLAIN*, R. C. BROWN, G. BERRY,
F. D. POOLEY†, R. DAVIES AND D. M. GRIFFITHS

*From the MRC Pneumoconiosis Unit, Llandough Hospital, Penarth, S. Glam. CF6 1XW
and the †Department of Mineral Exploitation, University College, Cardiff*

Received 12 August 1981 Accepted 10 November 1981

**Summary.—Tremolite is an amphibole which has been implicated in a variety of disease patterns in different parts of the world. It occurs in a number of phases, which are chemically identical but have specific physical characteristics. In an attempt to clarify the epidemiological findings, tremolite fibres of 3 specific forms—A, B and C—were characterized and studied for biological activity by:**

**(i) *in vivo* intrapleural injection of rats (2 separate experiments—1 with poor survival).**
**(ii) *in vitro* enzyme release from mouse peritoneal macrophages**
**(iii) *in vitro* giant-cell formation in A549 cultures**
**(iv) *in vitro* cytotoxicity for V79-4 cells.**

**Sample C, which contained more long thin fibres than A and B, was alone in producing mesotheliomas. C, but not A or B, induced LDH and B-glucuronidase enzyme release, and induced giant cells. A was not cytotoxic, B moderately cytotoxic and C as highly cytotoxic as UICC crocidolite.**

**The *in vivo* studies were marred by being split between 2 experiments, of which the second had poor survival.**

**We are aware of the weakness of our *in vivo* data, but as Tremolite C was being considered for commercial use on the European market we felt it timely to submit our findings for publication.**

TREMOLITE is an amphibole mineral (a chain silicate similar to asbestos) found in several countries. It has limited industrial value, but is used for stuccoing the exterior of buildings in the Middle East. It is frequently found as a contaminant of other minerals that are being exploited commercially.

Data on the health hazards of tremolite are currently being collected because it is an amphibole mineral and may be capable of causing diseases similar to those induced by amphibole asbestos (Wagner, 1980, 1982). A flake-like tremolite is found as a contaminant of talc in California but so far there is no evidence of disease which may be attributed to this tremolite. In the massive chrysotile ore bodies in Quebec Province in Canada, there are irregular deposits of a coarse-fibred tremolite. This material is found in the lungs of miners with pulmonary fibrosis and pleural plaques, but there is no correlation with mesotheliomas (Pooley, 1976). Further south in the northern part of New York State, a finer tremolite occurs as a contaminant of the talc deposits which are being exploited. From these mines there is evidence of pulmonary fibrosis, excess carcinoma of lung and pleura and a peritoneal mesothelioma (Kleinfeld *et al.*, 1967, 1974). Pleural plaques have occurred in the agricultural areas of Czechoslovakia and Yugoslavia and in the tobacco-growing regions of Bulgaria and Greece, all areas in which coarse tremolite fibres

* Present address: Unilever Research, Colworth House, Sharnbrook, Bedford, U.K.

> 1 μm in diameter are found in the soil. In Cyprus, a few mesotheliomas have occurred among the people living in the vicinity of the asbestos mine. This mine has large chrysotile ore bodies associated with tremolite, some of which is fine-fibred. These people use the tremolite, which is separated from the chrysotile during the milling process, for stuccoing their houses. A similar situation exists in Turkey, where in some areas the tremolite used for stuccoing is associated with the incidence of pleural calcification, mesotheliomas and bronchial cancers (Yazicioglu *et al.*, 1980). A pure tremolite consisting of fine fibres is mined in South Korea.

The carcinogenic potential of mineral dusts can be assessed by depositing the dusts in the pleural cavities of rats, either by injection (Wagner & Berry, 1969) or implantation (Stanton & Wrench, 1972). Previous work has demonstrated that the ability of a dust to induce mesotheliomas is related to the content of fibres over ~8 μm long and thinner than ~1·5 μm (Stanton *et al.*, 1977). Mammalian cells in tissue culture have been shown to be sensitive to the toxic effects of fibres of similar dimensions to those which are carcinogenic *in vivo* (Brown *et al.*, 1978; Chamberlain *et al.*, 1979; Wade *et al.*, 1980). It was considered important to test 3 types of tremolite which would cover the spectrum of particle types found throughout the world, using both *in vivo* and *in vitro* tests as the first stage in assessing the potential health hazards.

### MATERIALS AND METHODS

### *Dust samples*

The three samples of tremolite available were the flake-like material from the Californian talc deposits, a medium-sized fibrous mineral from Greenland and the fine-fibred material from South Korea. The samples used in this investigation were selected because upon degradation they were found to form fibrous particles with very different size distributions both in length and diameter ranges. All samples were prepared by milling in a small agate mill and ultrasonic dispersion, large particles being removed by sedimentation in water.

Tremolite Sample A was prepared from a sample of Californian tremolitic talc which originally contained 62% talc and 38%

FIG. 1.—Length and diameter distribution of fibres in Tremolite Sample A.

TABLE I.—*Particles/μg tremolite samples*

| Sample | No. of non-fibrous particles (× $10^4$) | Total no. of fibres (× $10^4$) | No. of fibres > 8 μm long (× $10^3$) and < 1·5 μm diameter |
|---|---|---|---|
| A | 6·9 | 5·1 | 1·7 |
| B | 20·7 | 4·8 | 0 |
| C | 3·3 | 15·5 | 56·1 |

tremolite. The talc in the sample was reduced by froth flotation to produce a tremolite sample >95% pure, the remaining material consisting mainly of talc together with minor magnesium and calcium carbonate material. The length and diameter distribution of the fibrous particles in this sample are illustrated by Fig. 1; most fibres were <6 μm long and <0·8 μm diameter. The number of particles/μg is shown in Table I. The chemical analysis is contained in Table II, from which it can be seen that the iron content of this tremolite is relatively low, but its distinguishing feature was that it contained significantly more potassium and sodium than the other samples.

Sample B was prepared from a tremolite rock specimen which originated from Greenland. On comminution the specimen was found to break down into fibrous particles, most of which were <3 μm long and <1·2 μm diameter (Fig. 2). The number of particles/μg is shown in Table I. In comparison with Tremolite A, this sample contained fibres which were on average both shorter and thicker. Sample B contained larger proportions of calcium and iron than Sample A and little potassium (Table II).

Sample C was prepared from a rock specimen which originated from South Korea. In appearance, the hand specimen contained no visual impurities, and on size reduction produced a dust which contained fibres up to 140 μm long, of which most were <0·6 μm diameter (see Fig. 3). The fibres in sample C were very much longer and finer than those in samples A and B. The number of particles/μg is presented in Table I. The iron content of Sample C was lower than that of Sample B but higher than that of Sample A (Table II).

The chemical compositions of the 3 samples are very similar, the exceptions being the K and Na in Sample A, and the Fe in Sample B.

*Analysis of fibre size distributions*

The methods used for the preparation of mineral dust samples for viewing in the transmission electronmicroscope (TEM) have been described fully elsewhere (Brown *et al.*, 1978). Briefly, an appropriately diluted sus-

FIG. 2.—Length and diameter distribution of fibres in tremolite Sample B.

TABLE II.—*Oxide composition (g/100 g) of tremolite samples (water of crystallization not included)*

| Sample | $Na_2O$ | MgO | $Al_2O_3$ | $SiO_2$ | $K_2O$ | CaO | FeO |
|---|---|---|---|---|---|---|---|
| A | 3·0 | 24·5 | 1·2 | 59·8 | 1·1 | 9·6 | 0·3 |
| B | 0·5 | 23·9 | 0·3 | 59·3 | 0·1 | 13·6 | 2·0 |
| C | 0·6 | 24·9 | 0·4 | 58·8 | 0·2 | 13·9 | 0·9 |

pension of each dust was filtered on to a 0·1 µm-pore-size Millipore membrane filter. The filters were coated with carbon and placed over parallel-bar EM grids which were on an acetone-soaked sponge. As the membrane filters dissolved, the carbon coat, and the dust particles, were deposited on the grids which were then viewed by TEM. Overlapping fields were photographed and large mosaics constructed. The length and diameter of at least 300 fibres were measured where possible, and the numbers of fibres and non-fibrous particles determined. The rules advocated by Cooper *et al.* (1978) were used for counting the fibres on the photographs. Typical photographs of each dust are shown in Fig. 4.

*In vivo carcinogenicity*

The experimental animals used in this investigation were barrier-protected SPF rats of the Sprague–Dawley and Wistar strains. Each dust sample was prepared in physiological saline (0·9% w/v NaCl in distilled water) at 50 mg/ml and sterilized by autoclaving (15 lb/in² for 20 min). The dose of 20 mg of experimental material per rat was injected into the right pleural cavity (Wagner & Berry, 1969); animals receiving 0·4 ml saline served as controls. Equal numbers of males and females were used in each experimental group.

Sample A was injected into 32 Wistar rats 2 years before the rest of the investigation.

FIG. 3.—Length and diameter distribution of fibres in Tremolite Sample C.

FIG. 4.—Electron micrographs of the 3 samples of tremolite.

Rats receiving SFA chrysotile served as positive controls in this experiment.

Samples B and C were injected into groups of 48 Sprague–Dawley rats; a group of 32 animals receiving UICC crocidolite served as positive controls.

Rats were 8–10 weeks old when injected and were allowed to live out their lives.

In vitro *toxicity*

*Enzyme release from mouse peritoneal macrophages.*—Mouse peritoneal macrophages were obtained from 22–27g Swiss TO mice (Tuck and Son Ltd, Battlebridge, Essex) by peritoneal lavage using 3·5 ml Medium 199 containing 5 i.u. heparin, 100 i.u. benzylpenicillin and 100 μg streptomycin per ml. About $1{\cdot}5 \times 10^6$ cells in 2 ml of the above medium were placed in albumin-coated 35mm-diameter Petri dishes (Davies, 1980) and allowed to attach for 1 h at 37°C. The non-adherent cells were then removed by washing with phosphate-buffered saline. The remaining cells were cultured in 2 ml Medium 199 containing antibiotics and 10% heat-inactivated acid-treated foetal calf serum (Chamberlain *et al.*, 1979) in an atmosphere of 5% $CO_2$ in air at 37°C.

The medium of macrophage cultures prepared 24 h previously was replaced by 2 ml of medium containing the test dust at 50, 100 or 150 μg/ml; control cultures received medium without dust. After 18h incubation the medium was collected and the adherent cells disrupted by the addition of 2 ml saline containing 0·1% Triton X-100 and 0·1% bovine serum albumin and rubbing the Petri-dish surface with a silicone-rubber bung. Both the medium and the cell lysates were centrifuged at 800 *g* for 10 min and the supernatants assayed for lactic dehydrogenase (LDH) and β-glucuronidase (BGL) by the continuous-flow fluorimetric method of Morgan *et al.* (1978) using a Perkin Elmer Model 3000 fluorescence spectrophotometer.

*Giant-cell formation in A549 cultures.*—Type II alveolar cells (A549) derived from a human tumour (Leiber *et al.*, 1976) were obtained from Dr G. Todaro, NCI, Bethesda, Maryland, U.S.A. The cells were grown in Dulbecco's modification of Eagle's minimal essential medium supplemented with 10% heat-inactivated FCS and antibiotics in an atmosphere of 10% $CO_2$ in air at 37°C.

A standard inoculum of $10^5$ cells was added to each of a series of 25cm² culture flasks along with an appropriate amount of dust suspension. Four flasks were used for each dust, 2 at a dust concentration of 100 μg/ml and 2 at 200 μg/ml. Flasks with no dust served as controls. All the cultures were incubated for 5 days, the cells detached using trypsin–EDTA and suspended in an appropriate volume of medium and photographed on a haemacytometer. The diameters of 200 cells from each treatment were measured as described in Chamberlain & Brown (1978).

*Cytotoxicity to V79-4 cells.*—Chinese hamster lung cells (V79-4) described by Chu & Malling (1968) were obtained from Dr C. F. Arlett, MRC Cell Mutation Unit, Brighton, and cultured in MEM supplemented with 15% FCS and antibiotics at 37°C in an atmosphere of 5% $CO_2$ in air.

This method has been reported in detail elsewhere (Chamberlain & Brown, 1978). Briefly, the survival of V79-4 cells in the absence or presence of a series of concentrations of each dust was determined by adding the appropriate amount of dust to a suspension of single cells. The cell/dust mixtures were then placed on 60mm-diameter Petri dishes (~200 cells/dish) and incubated for 6 days. After incubation the medium was removed, the cells fixed with 10% formal saline and stained with 1% methylene blue. The colonies on each dish were counted in an automatic colony counter (Micro Measurements Ltd, Cambridge).

### RESULTS

### *Physical characteristics of the dusts*

All of the dusts contained fibres; representative photographs are shown in Fig. 4. The size distributions of the fibres in each dust are shown in Figs 1–3 and the numbers of particles per μg are presented in Table I. Samples A and B contained relatively few fibres and Sample C contained many very long thin fibres.

### *Induction of mesotheliomas*

The percentages of rats developing a mesothelioma following the various treatments are shown in Table III. The survival of the animals in Experiment II was poor because of infection, and is discussed later.

### In vitro *toxicity*

*Enzyme release from mouse peritoneal macrophages.*—The release of both LDH and BGL from mouse peritoneal macrophages after 18h incubation with each of the dusts is shown in Table IV. Samples A and B had little effect on the cells, Sample C induced the release of 30% LDH and over 60% BGL.

*Giant-cell formation in A549 cells.*—The percentage of giant cells induced by each dust in cultures of A549 cells is shown in Table V. UICC crocidolite induced a significant percentage of giant

TABLE III.—*Carcinogenic activities of the dusts in experimental animals*

| | No. of rats examined | Mean survival after injection (days) | mesotheliomas (%) |
|---|---|---|---|
| *Expt I* | | | |
| Saline control | 32 | 717 | 0 (0) |
| Sample A | 31 | 644 | 0 (0) |
| SFA chrysotile (+ve control) | 32 | 612 | 20 (62) |
| *Expt II* | | | |
| Saline control | 23 | 552 | 0 (0) |
| Sample B | 48 | 549 | 0 (0) |
| Sample C | 47 | 541 | 14 (30) |
| UICC crocidolite (+ve control) | 31 | 557 | 2 (6) |

TABLE IV.—*Activity of dusts against mouse peritoneal macrophages, measured by release of enzymes (mean of* 4 *cultures* ± 95% *confidence limits)*

| Dust at 100 μg/ml | % LDH release | % BGL-release |
|---|---|---|
| Control | 5·6±0·4 | 3·6±0·3 |
| Tremolite A | 10·4±1·2 | 9·9±0·8 |
| Tremolite B | 14·9±0·8 | 14·9±2·6 |
| Tremolite C | 28·5±0·9 | 62·8±1·0 |
| UICC crocidolite | 39·1±1·7 | 48·5±4·5 |

TABLE V.—*Activity of dusts against A*549 *cells (% of giant cells, with* 95% *confidence limits; giant cells defined as those* > 25 *μm diameter)*

| Treatment | | |
|---|---|---|
| Control | 1·47 (0·5–4·2) | |
| Dose | 100 μg/ml | 200 μg/ml |
| Tremolite A | 1·0 (0·3–3·6) | 4·5 (2·4–8·3) |
| Tremolite B | 5·3 (3·0–9·2) | 3·0 (1·3–6·9) |
| Tremolite C | 19·8 (14·9–25·8) | 24·5 (19·1–30·9) |
| UICC crocidolite | 14·4 (10·2–19·9) | 26·3 (20·1–32·7) |

TABLE VI.—*Cytotoxicity to V79-4 cells*

| Dust | % survival at 50 μg/ml (± 95% confidence limits) |
|---|---|
| Tremolite A | 101·0±11·5 |
| Tremolite B | 36·7±6·7 |
| Tremolite C | 3·5±1·2 |
| UICC Crocidolite | 2·9±1·2 |

cells, as reported by Chamberlain & Brown (1978). Of the test dusts, only Sample C induced giant cells, and it was as active as UICC crocidolite.

*Cytotoxicity to V*79-4 *cells*.—The cytotoxic potentials of the dusts towards V79-4 cells are shown in Table VI. Sample A was inert, B was moderately toxic, but C was as toxic as UICC crocidolite.

DISCUSSION

As indicated in the introduction, data on the human health hazards of tremolite are currently being collected. We report here experimental studies on both the carcinogenic effects *in vivo* and the cytotoxic effect *in vitro*, of 3 samples of tremolite.

Many inorganic dusts have been shown to be carcinogenic in experimental animals (for a review see IARC, 1977). Stanton *et al.* (1977) and Stanton & Layard (1978) demonstrated that the carcinogenic potential of a dust correlates with the number of fibres longer than ~8 μm and thinner than ~1·5 μm per unit mass. We have reported previously that fibres of very similar size are responsible for cytotoxic effects in 3 types of mammalian cells (Brown *et al.*, 1978; Chamberlain *et al.*, 1979). Wade *et al.* (1980) have made similar observations. In view of the fact that fibres of similar size are both carcinogenic *in vivo* and cytotoxic *in vitro*, the use of certain mammalian cells for the detection of potentially pathogenic dusts has been proposed (Chamberlain *et al.*, 1979; Wade *et al.*, 1980; Brown *et al.*, 1980).

Only one of the tremolite samples, C, was carcinogenic. This sample was also consistently very active in the 3 *in vitro* systems. Sample C contained $5{\cdot}6 \times 10^4$ fibres $>8$ $\mu$m long and $<1{\cdot}5$ $\mu$m in diameter per $\mu$g. UICC crocidolite, used as a carcinogen-positive control dust, contained $6{\cdot}4 \times 10^4$ fibres of this size per $\mu$g. UICC crocidolite and Tremolite Sample C were found to be very similar in their activities in the *in vitro* systems (Tables IV, V & VI). However, Tremolite Sample C seemed to be more carcinogenic than UICC crocidolite (Table III).

It is a weakness that the animal data reported here though from 2 separate experiments, using 2 strains of rat, were impaired by the poor survival due to infection in the second experiment. In this experiment only 2 Sprague–Dawley rats (6%) injected with the UICC corcidolite positive control developed mesotheliomas. This is much lower than obtained previously with Wistar rats (Wagner *et al.*, 1973; Berry & Wagner, 1976; Wagner *et al.*, 1980*a*) which gave 46% mesotheliomas on average. The mean survival in these earlier experiments was over 4 months longer than in the experiment reported here, which partly explains the difference in mesothelioma rate. However, after allowing for survival, the mesothelioma rate in the second experiment reported here was only between 1/4 and 1/2 of the previous rates. The reason for this low rate is unknown, but an obvious possibility is that it is characteristic of the Sprague–Dawley strain that we used. However, in another experiment carried out during the same period, 6 Sprague–Dawley rats out of 48 developed mesotheliomas after injections with UICC African chrysotile (Wagner *et al.*, 1980*b*) with a mean survival only one month longer than with crocidolite in the experiment reported here. In Wistar rats UICC crocidolite produces more mesotheliomas than UICC chrysotile (Wagner *et al.*, 1973). Thus a low mesothelioma rate is not characteristic of the Sprague–Dawley strain that we used. A second possibility is that the low rate with crocidolite was a chance finding, and comparison of the present experiment with the earlier ones indicates that this possibility cannot be excluded ($P > 0{\cdot}1$). The present experiment is imprecise due to the poor survival; in terms of mesothelioma rate, there is an efficiency of only 40% of the earlier ones, *i.e.* the 31 rats with low survival are equivalent to only 12 rats with the longer survival previously obtained with Wistar rats.

Unsatisfactory though the experiment on carcinogenesis of Sample C may be, owing to the near failure of the positive controls, the fact remains that Sample C produced 14 mesotheliomas in 47 rats, whereas Samples A and B produced none.

In view of the foregoing remarks we think that it is wiser to interpret the data presented here in a qualitative rather than a quantitative manner. By analogy with other members of the amphibole asbestos minerals we suspect that Tremolite Sample C, originating from South Korea, would be a human health hazard if present in sufficient airborne concentration. An experiment exposing rats to airborne clouds of this tremolite by inhalation is now being planned as the next stage in assessing the potential health hazard.

### REFERENCES

Berry, G. & Wagner, J. C. (1976) Effect of age at inoculation of asbestos on occurrence of mesotheliomas in rats. *Int. J. Cancer*, **17**, 477.

Brown, R. C., Chamberlain, M., Griffiths, M. & Timbrell, V. (1978) The effect of fibre size on the *in vitro* biological activity of three types of amphibole asbestos. *Int. J. Cancer*, **22**, 721.

Brown, R. C., Chamberlain, M., Davies, R. & Sutton, G. T. (1980) The *in vitro* activities of pathogenic mineral dusts. *Toxicology*, **17**, 143.

CHAMBERLAIN, M. & BROWN, R. C. (1978) The cytotoxic effects of asbestos and other mineral dust in tissue culture cell lines. *Br. J. Exp. Pathol.*, **59**, 183.

CHAMBERLAIN, M., BROWN, R. C., DAVIES, R. & GRIFFITHS, D. M. (1979) *In vitro* prediction of the pathogenicity of mineral dusts. *Br. J. Exp. Pathol.*, **60**, 320.

CHU, E. H. Y. & MALLING, H. V. (1968) Mammalian cell genetics: Chemical induction of specific locus mutations in Chinese Hamster cells *in vitro*. *Proc. Natl Acad. Sci.*, **61**, 1306.

COOPER, D. W., FELDMAN, H. A. & CHASE, G. R. (1978) Fiber counting: A source of error corrected. *Am. Ind. Hyg. Assoc. J.*, **39**, 362.

DAVIES, R. (1980) The effect of dusts on enzyme release from macrophages. In *The* In Vitro *Effects of Mineral Dusts*, (Eds Brown *et al.*) London: Academic Press. p. 67.

IARC (1977) The Evaluation of Carcinogenic Risk of Chemicals to Man. 14, Asbestos. Lyon: IARC.

KLEINFELD, M., MESSITE, J., KOOYMAN, O. & ZAKI, M. H. (1967) Mortality among talc miners and millers in New York State. *Arch. Environ. Hlth*, **14**, 663.

KLEINFELD, M., MESSITE, J. & ZAKI, M. H. (1974) Mortality experiences among talc workers: A follow-up study. *J. Occup. Med.*, **16**, 345.

LEIBER, M., SMITH, S., SZAKAL, A., NELSON-REES, W. & TODARO, G. (1976) A continuous tumour-cell line from a human lung carcinoma with properties of Type II alveolar epithelial cells. *Int. J. Cancer*, **17**, 62.

MORGAN, D. M. L., VINT, S. & RIDEOUT, J. M. (1978) Continuous flow fluorimetric assay of lysosomal enzymes. *Med. Lab. Sci.*, **35**, 335.

POOLEY, F. D. (1976) An examination of the fibrous mineral content of asbestos lung tissue from the Canadian chrysotile mining industry. *Environ. Res.*, **12**, 281.

STANTON, M. F. & WRENCH, C. (1972) Mechanisms of mesothelioma induction with asbestos and fibrous glass. *J. Natl Cancer Inst.*, **48**, 797.

STANTON, M. F., LAYARD, M., TEGERIS, A., MILLER, E., MAY, M. & KENT, E. (1977) Carcinogenicity of fibrous glass: pleural response in the rat in relation to fiber dimensions. *J. Natl Cancer Inst.*, **58**, 587.

STANTON, M. F. & LAYARD, M. (1978) The carcinogenicity of fibrous minerals. In *Proceedings of a Workshop on Asbestos: Definitions and Measurement Methods.* (Eds Gravatt *et al.*) National Bureau of Standards (U.S.A.) Special Publication, **506**, 143.

WADE, M. J., LIPKIN, L. E., STANTON, M. F. & FRANK, A. L. (1980) P388$D_1$ *in vitro* cytotoxicity assay as applied to asbestos and other minerals: Its possible relevance to carcinogenicity. In *The* in Vitro *Effects of Mineral Dusts.* (Eds Brown *et al.*) London: Academic Press. p. 351.

WAGNER, J. C. (1980) Environmental and occupational exposure to natural mineral fibres. In *Biological Effects of Mineral Fibres.* (Ed. Wagner.) Lyon: IARC. p. 995.

WAGNER, J. C. (1982) *Mineral Fibre Carcinogenesis.* Am. Chem. Soc. Monogr. (in the press).

WAGNER, J. C. & BERRY, G. (1969) Mesotheliomas in rats following inoculation with asbestos. *Br. J. Cancer*, **23**, 567.

WAGNER, J. C., BERRY, G. & TIMBRELL, V. (1973) Mesotheliomata in rats after inoculation with asbestos and other materials. *Br. J. Cancer*, **28**, 173.

WAGNER, J. C., HILL, R. J., BERRY, G. & WAGNER, M. M. F. (1980*a*) Treatments affecting the rate of asbestos-induced mesotheliomas. *Br. J. Cancer*, **41**, 918.

WAGNER, J. C., BERRY, G., HILL, R. J., MUNDAY, D. E. & SKIDMORE, J. W. (1980*b*) Animal experiments with man-made mineral fibres. In *The Biological Effects of Mineral Fibres.* (Ed. Wagner). Lyon: IARC. p. 361.

YAZICIOGLU, S., IKAYTO, R., BALCI, K., SAYLI, B. S. & YORULMAZ, B. (1980) Pleural calcification, pleural mesotheliomas and bronchial cancers caused by tremolite dust. *Thorax*, **35**, 564.


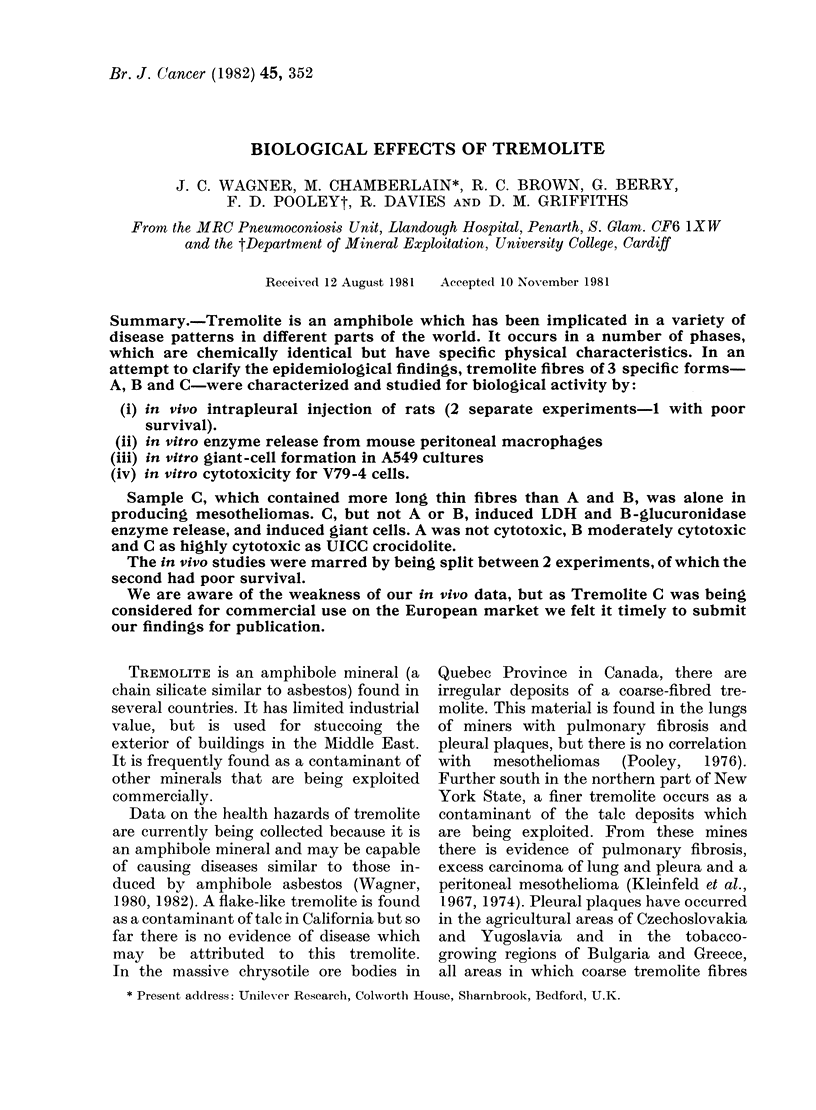

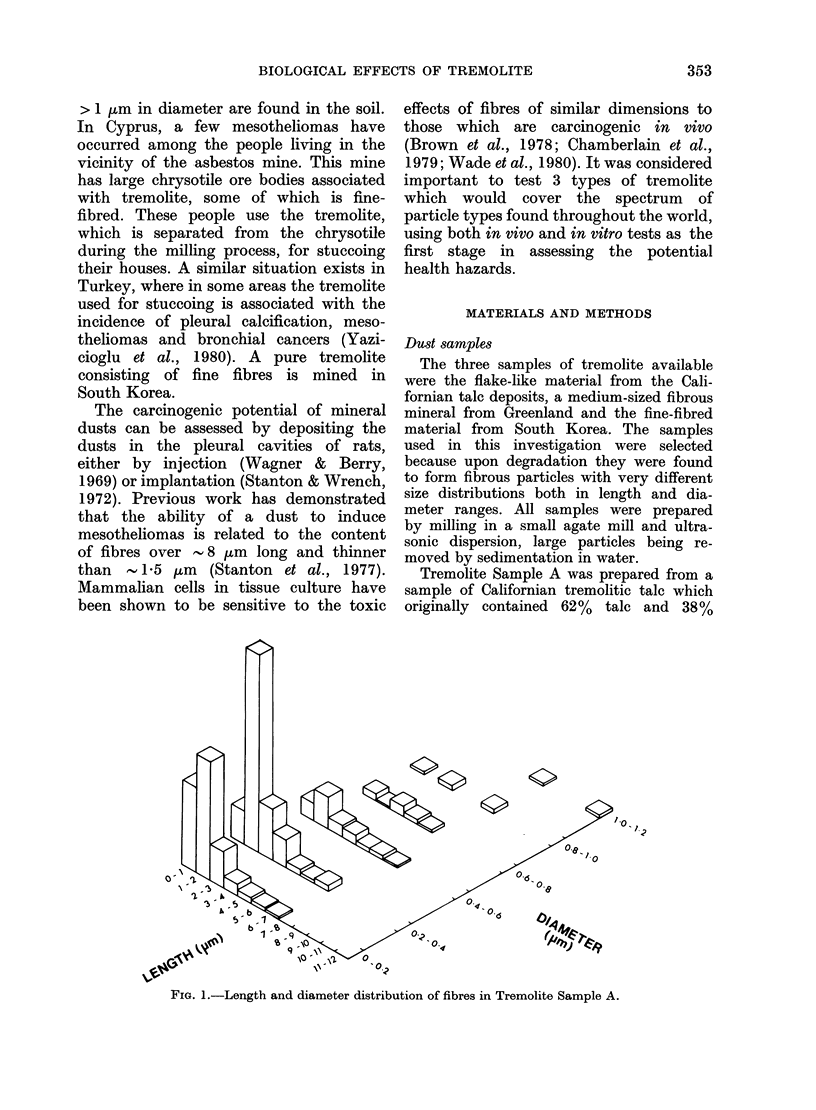

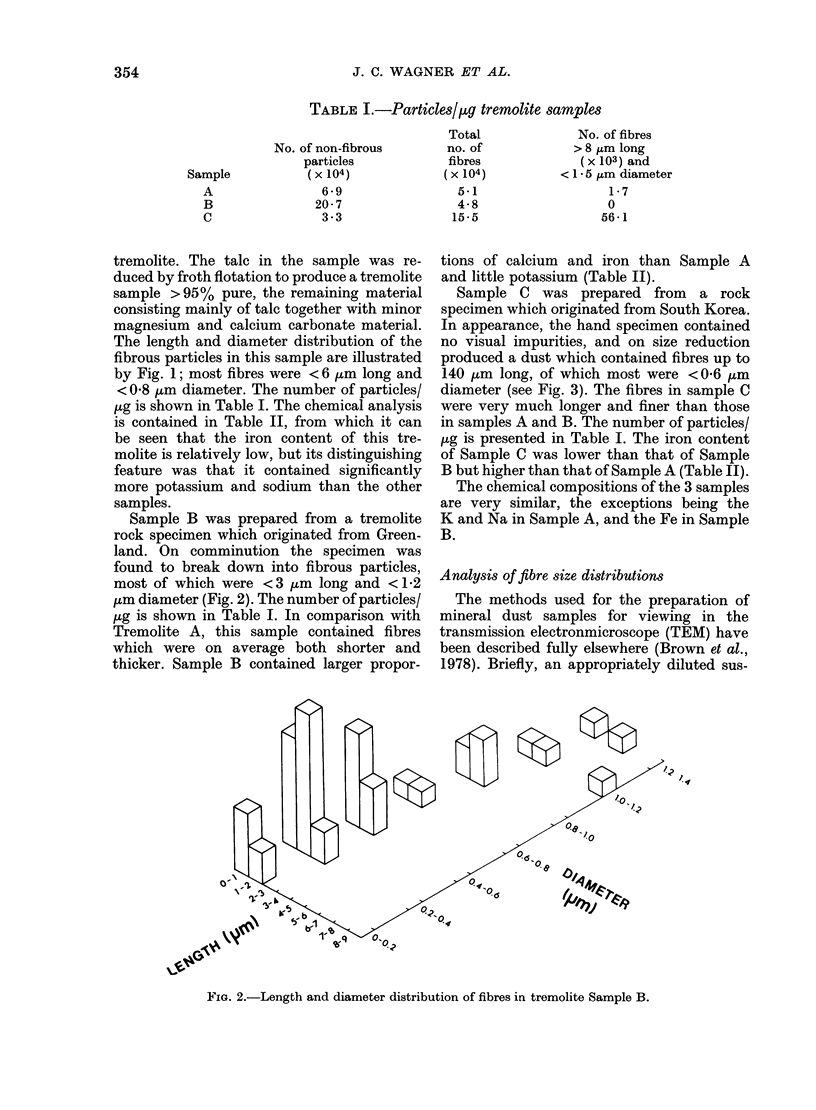

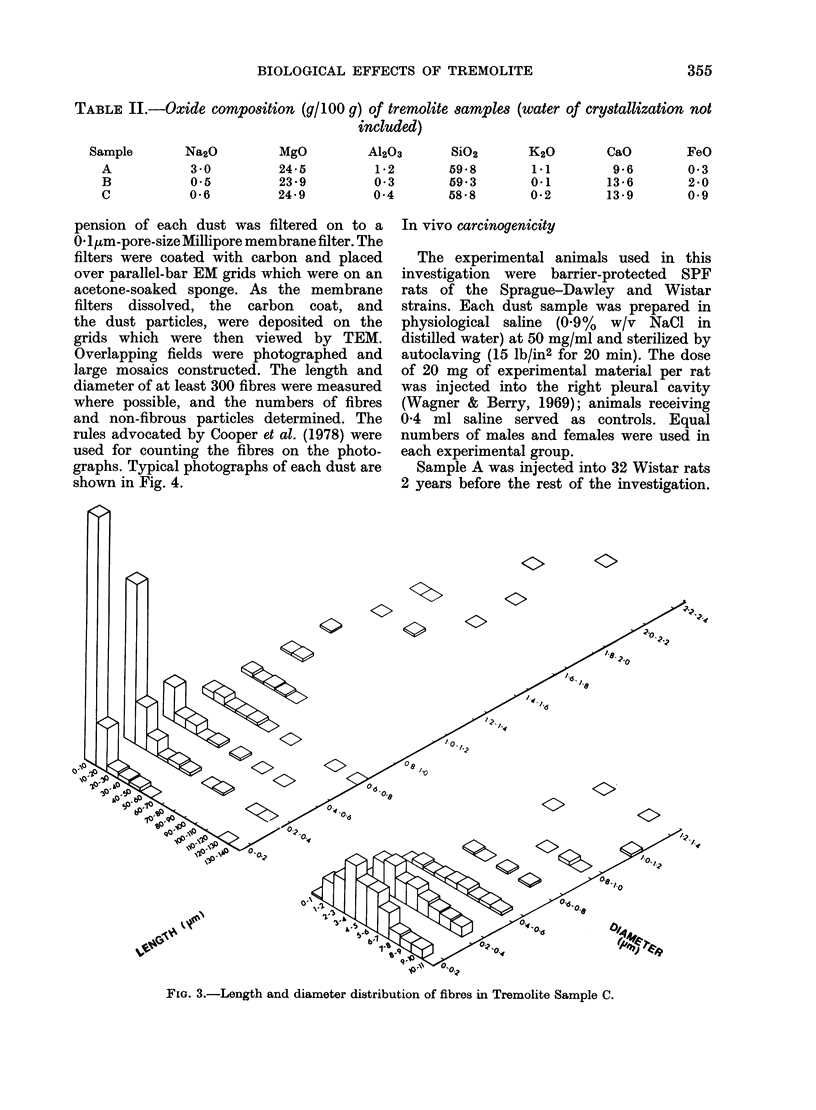

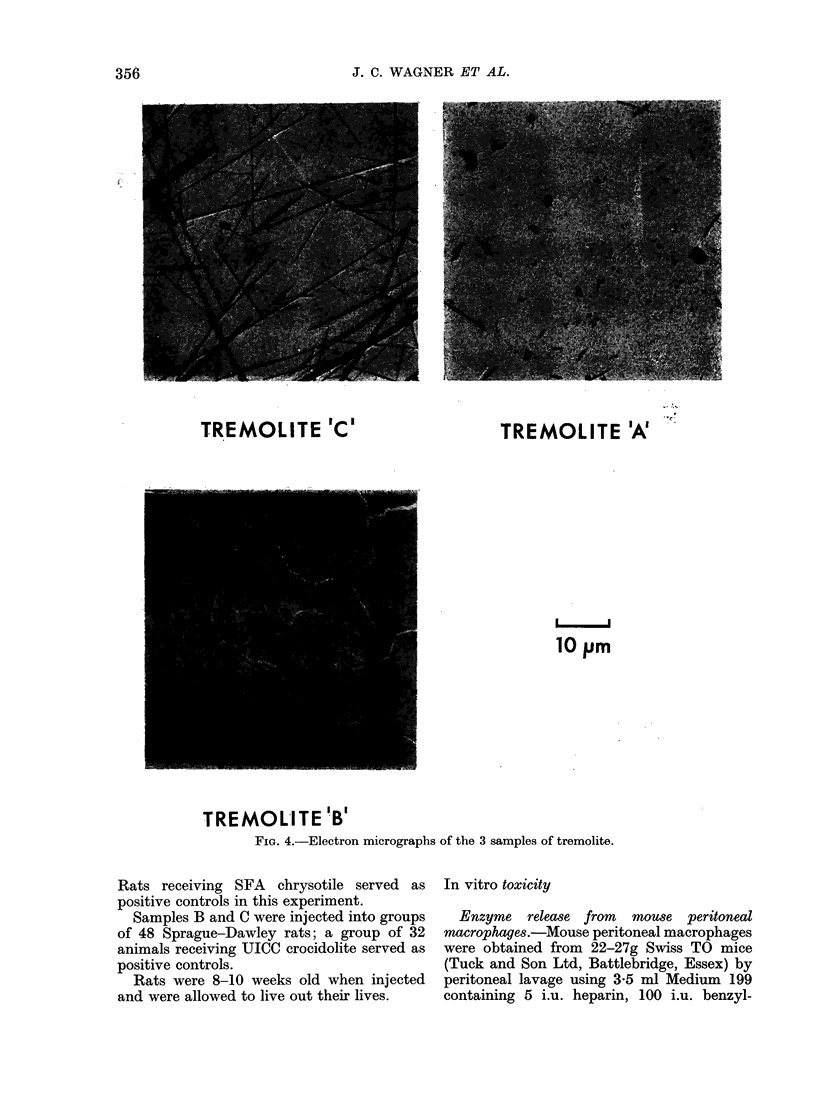

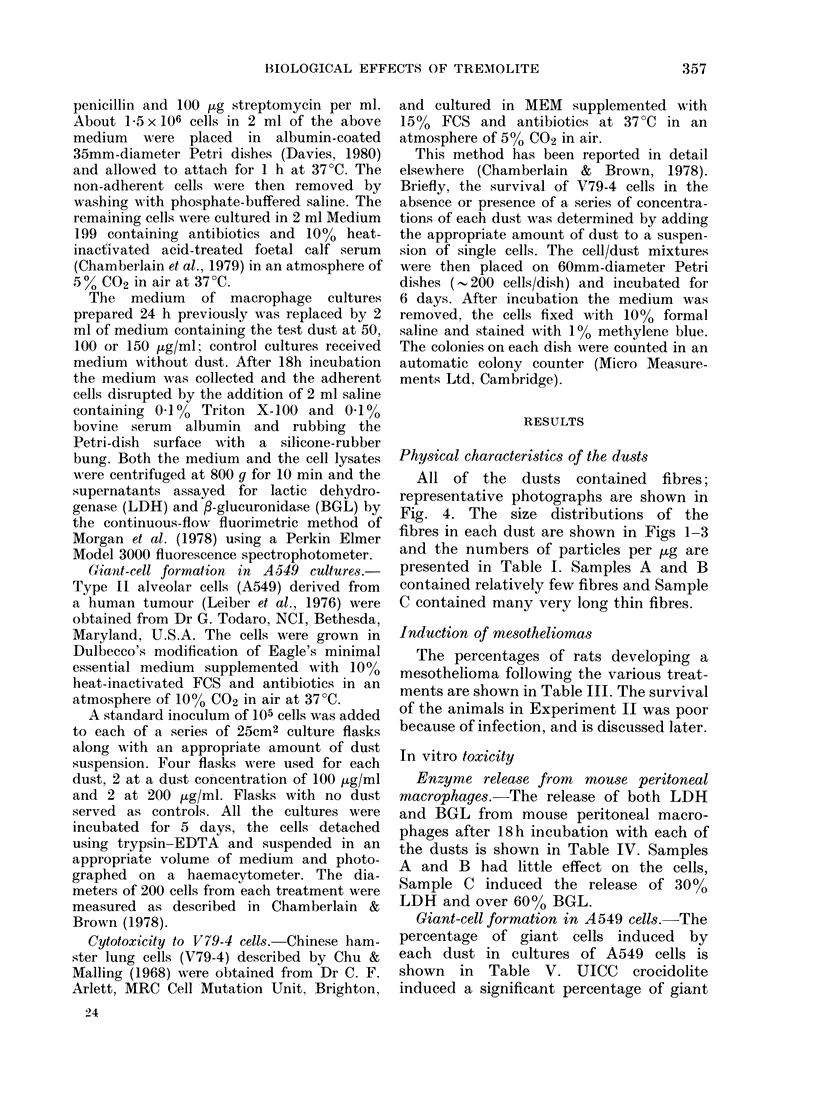

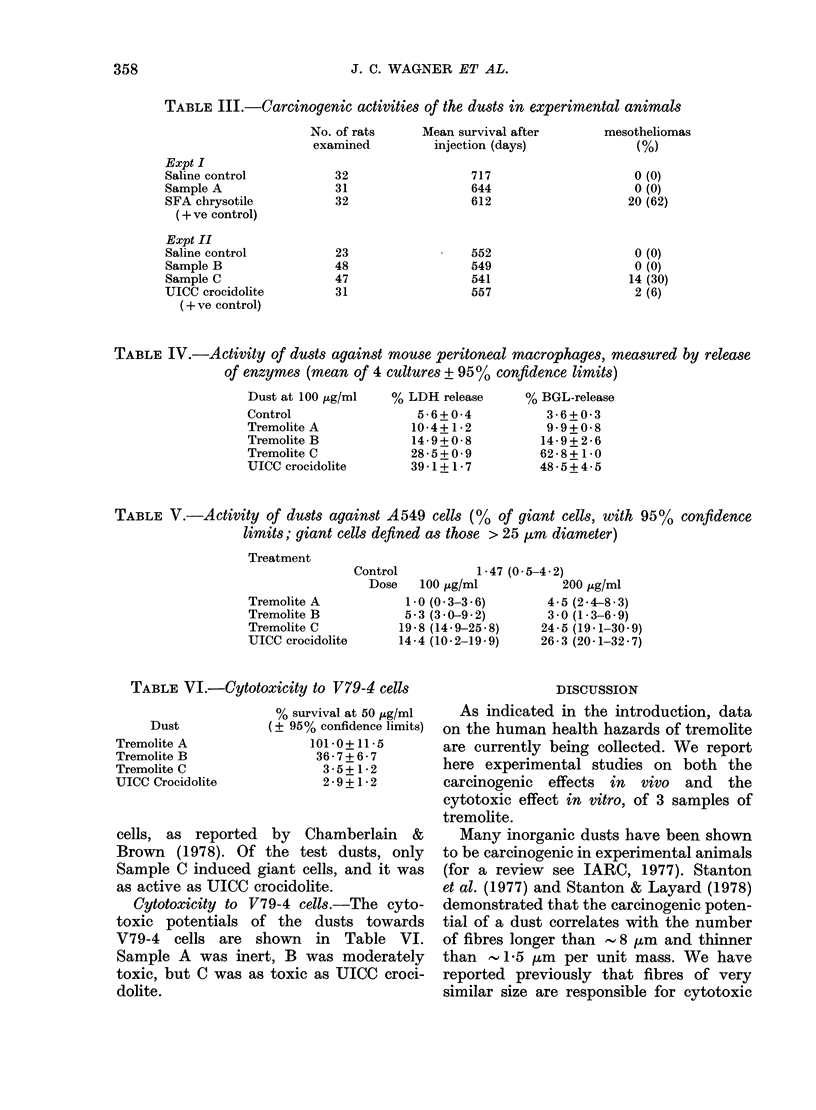

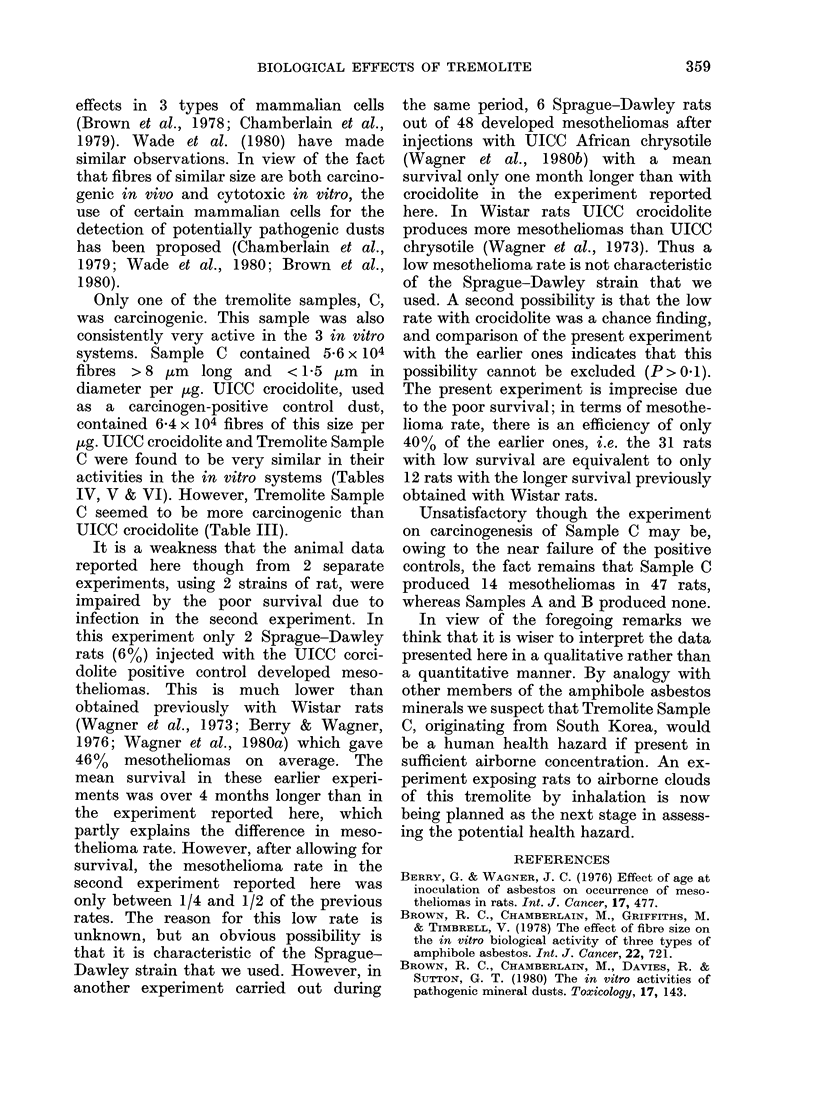

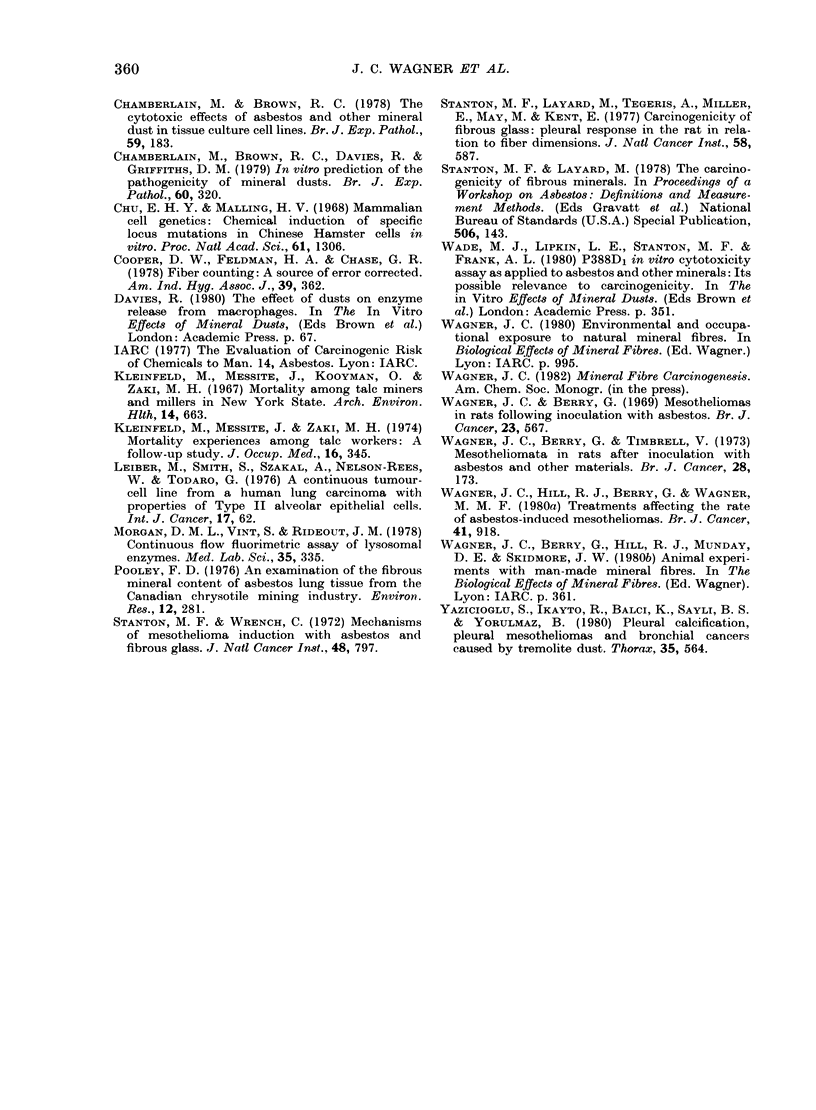


## References

[OCR_00792] Berry G., Wagner J. C. (1976). Effect of age at inoculation of asbestos on occurrence of mesotheliomas in rats.. Int J Cancer.

[OCR_00803] Brown R. C., Chamberlain M., Davies R., Sutton G. T. (1980). The in vitro activities of pathogenic mineral dusts.. Toxicology.

[OCR_00797] Brown R. C., Chamberlain M., Griffiths D. M., Timbrell V. (1978). The effect of fibre size on the in vitro biological activity of three types of amphibole asbestos.. Int J Cancer.

[OCR_00816] Chamberlain M., Brown R. C., Davies R., Griffiths D. M. (1979). In vitro prediction of the pathogenicity of mineral dusts.. Br J Exp Pathol.

[OCR_00810] Chamberlain M., Brown R. C. (1978). The cytotoxic effects of asbestos and other mineral dust in tissue culture cell lines.. Br J Exp Pathol.

[OCR_00822] Chu E. H., Malling H. V. (1968). Mammalian cell genetics. II. Chemical induction of specific locus mutations in Chinese hamster cells in vitro.. Proc Natl Acad Sci U S A.

[OCR_00828] Cooper D. W., Feldman H. A., Chase G. R. (1978). Fiber counting: a source of error corrected.. Am Ind Hyg Assoc J.

[OCR_00843] Kleinfeld M., Messite J., Kooyman O., Zaki M. H. (1967). Mortality among talc miners and millers in New York State.. Arch Environ Health.

[OCR_00849] Kleinfeld M., Messite J., Zaki M. H. (1974). Mortality experiences among talc workers: a follow-up study.. J Occup Med.

[OCR_00854] Lieber M., Smith B., Szakal A., Nelson-Rees W., Todaro G. (1976). A continuous tumor-cell line from a human lung carcinoma with properties of type II alveolar epithelial cells.. Int J Cancer.

[OCR_00861] Morgan D. M., Vint S., Rideout J. M. (1978). Continuous flow fluorimetric assay of lysosomal enzymes.. Med Lab Sci.

[OCR_00866] Pooley F. D. (1976). An examination of the fibrous mineral content of asbestos lung tissue from the Canadian chrysotile mining industry.. Environ Res.

[OCR_00877] Stanton M. F., Laynard M., Tegeris A., Miller E., May M., Kent E. (1977). Carcinogenicity of fibrous glass: pleural response in the rat in relation to fiber dimension.. J Natl Cancer Inst.

[OCR_00872] Stanton M. F., Wrench C. (1972). Mechanisms of mesothelioma induction with asbestos and fibrous glass.. J Natl Cancer Inst.

[OCR_00910] Wagner J. C., Berry G. (1969). Mesotheliomas in rats following inoculation with asbestos.. Br J Cancer.

[OCR_00915] Wagner J. C., Berry G., Timbrell V. (1973). Mesotheliomata in rats after inoculation with asbestos and other materials.. Br J Cancer.

[OCR_00921] Wagner J. C., Hill R. J., Berry G., Wagner M. M. (1980). Treatments affecting the rate of asbestos-induced mesotheliomas.. Br J Cancer.

[OCR_00934] Yazicioglu S., Ilçayto R., Balci K., Sayli B. S., Yorulmaz B. (1980). Pleural calcification, pleural mesotheliomas, and bronchial cancers caused by tremolite dust.. Thorax.

